# Supplementary material for: Parallel gene amplification by Cas9 nickase for generating functionally heterogeneous cell populations
Source: Cell Rep Methods. 2026 May 25;6(7):101467. doi: 10.1016/j.crmeth.2026.101467 (PMC13389981; doi:10.1016/j.crmeth.2026.101467)
Supplement: Document S1. Figures S1 and S2 [file mmc1.pdf]

Cell Reports Methods, Volume *6*

Supplemental information

Parallel gene amplification by Cas9 nickase for generating functionally heterogeneous cell populations

Hiroaki Takesue, Satoshi Okada, and Takashi Ito

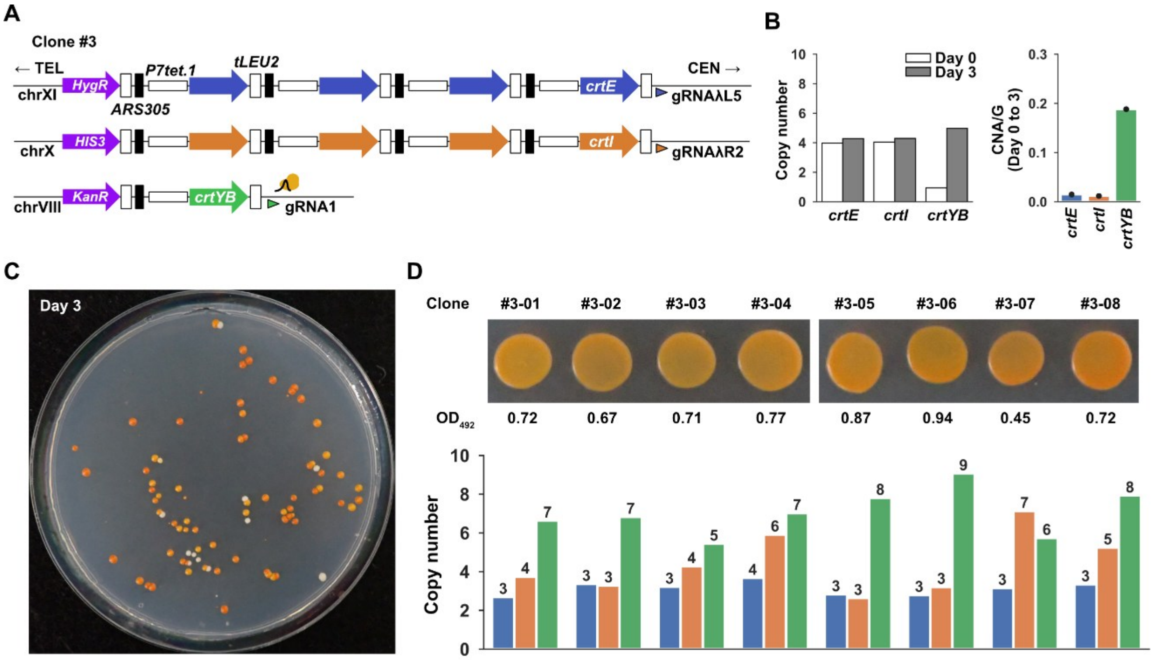

Figure S1. Independent generation of elite clones by simplex BITREx, related to Figure 4

(A) Genetic architecture of the parental strain clone #3. This parental strain had already acquired four copies each of *crtE* and *crtI* at the initial strain construction phase using the standard genome editing. Accordingly, only gRNA1 was expressed in clone #3 to induce the selective amplification of *crtYB*.

(B) Copy number alterations of carotenogenic genes. Clone #3 was subjected to simplex BITREx for *crtYB* in the presence of β-estradiol. Copy numbers of each target gene at day 0 and day 3 were quantified by qPCR (left and middle panels), and the corresponding CNA/G values were calculated (right panel).

(C) Colonies on Dox-containing agar plates. To cease BITREx and induce β-carotene synthesis, the 3-day culture of clone #3 was spread on agar plates containing Dox but lacking β-estradiol.

(D) Analysis of representative elite clones. The 3-day culture of clone #3 was spread on agar plates containing Dox but lacking β-estradiol to cease BITREx and induce β-carotene synthesis. Top panel: Representative eight clones are shown as patches alongside their corresponding OD_{492} values of the cell extract, which serve as a proxy for β-carotene yield. Bottom panel: Bar graphs indicate the copy numbers of the three carotenogenic genes in these eight clones, as estimated from nanopore sequencing data.

A

Clone	Day 0			Day 4			
	Copy number value			Generation	Copy number value		
	crtE	crtI	crtYB		crtE	crtI	crtYB
#1-01	10 (1.00)	1 (1.00)	BLQ (N.C.)	30.5	15 (1.41)	1 (1.31)	BLQ (N.C.)
#1-02	15 (1.00)	BLQ (N.C.)	5 (1.00)	30.4	15 (1.00)	BLQ (N.C.)	5 (1.06)
#1-03	3 (1.00)	BLQ (N.C.)	18 (1.00)	30.8	3 (0.92)	BLQ (N.C.)	13 (0.75)
#1-04	14 (1.00)	BLQ (N.C.)	3 (1.00)	28.6	16 (1.15)	BLQ (N.C.)	5 (1.67)
#1-05	32 (1.00)	18 (1.00)	1 (1.00)	30.8	20 (0.63)	18 (1.04)	1 (1.27)
#1-06	45 (1.00)	7 (1.00)	1 (1.00)	29.9	45 (1.01)	8 (1.09)	1 (1.62)
#1-07	25 (1.00)	1 (1.00)	2 (1.00)	28.6	27 (1.10)	1 (1.16)	3 (1.49)
#1-08	19 (1.00)	1 (1.00)	1 (1.00)	28.4	23 (1.21)	2 (2.73)	2 (2.39)
#1-09	12 (1.00)	15 (1.00)	6 (1.00)	28.1	12 (1.04)	17 (1.15)	9 (1.58)
#1-10	20 (1.00)	1 (1.00)	5 (1.00)	28.5	21 (1.05)	1 (1.51)	7 (1.44)
#1-11	9 (1.00)	1 (1.00)	10 (1.00)	28.3	16 (1.84)	2 (2.73)	19 (1.87)
#1-12	18 (1.00)	1 (1.00)	5 (1.00)	28.6	22 (1.18)	3 (3.65)	8 (1.76)
#1-13	16 (1.00)	4 (1.00)	10 (1.00)	30.5	15 (0.95)	8 (1.76)	12 (1.24)
#1-14	7 (1.00)	17 (1.00)	3 (1.00)	31.4	8 (1.12)	21 (1.21)	4 (1.33)
#1-15	13 (1.00)	37 (1.00)	9 (1.00)	31.2	15 (1.76)	29 (0.78)	11 (1.27)
#1-16	4 (1.00)	4 (1.00)	7 (1.00)	29.2	5 (1.21)	7 (1.70)	11 (1.50)

B

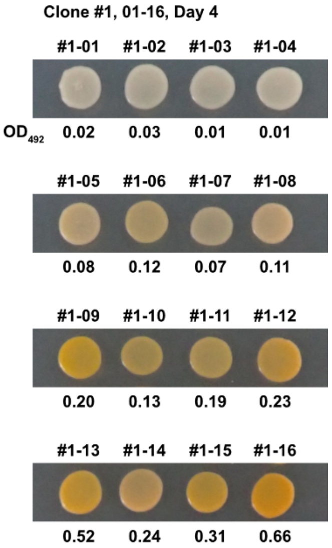

Figure S2. Stability of expanded arrays of carotenogenic genes, related to Figure 4

(A) Stability of copy numbers for the three carotenogenic genes during 4-day cultivation. Copy-number retention in the 16 clones (originally shown in Figure 4E) was monitored via qPCR during passaging without β-estradiol. The estimated generation numbers, derived from OD_{620} values, are provided for each time point. Values in parentheses represent relative copy numbers normalized to those at day 0. BLQ, below the lower limit of quantification; N.C., not calculated.

(B) Maintenance of production phenotypes. Cell spots of the 16 clones after 4-day cultivation on Dox-containing agar plates are shown alongside the corresponding OD_{492} values of the cell extracts.
