# Supplementary material for: Parallel gene amplification by Cas9 nickase for generating functionally heterogeneous cell populations
Source: Cell Rep Methods. 2026 May 25;6(7):101467. doi: 10.1016/j.crmeth.2026.101467 (PMC13389981; doi:10.1016/j.crmeth.2026.101467)
Supplement: Document S2. Article plus supplemental information [file mmc6.pdf]

Cell Reports
Methods

Article

Parallel gene amplification by Cas9 nickase for generating functionally heterogeneous cell populations

Graphical abstract

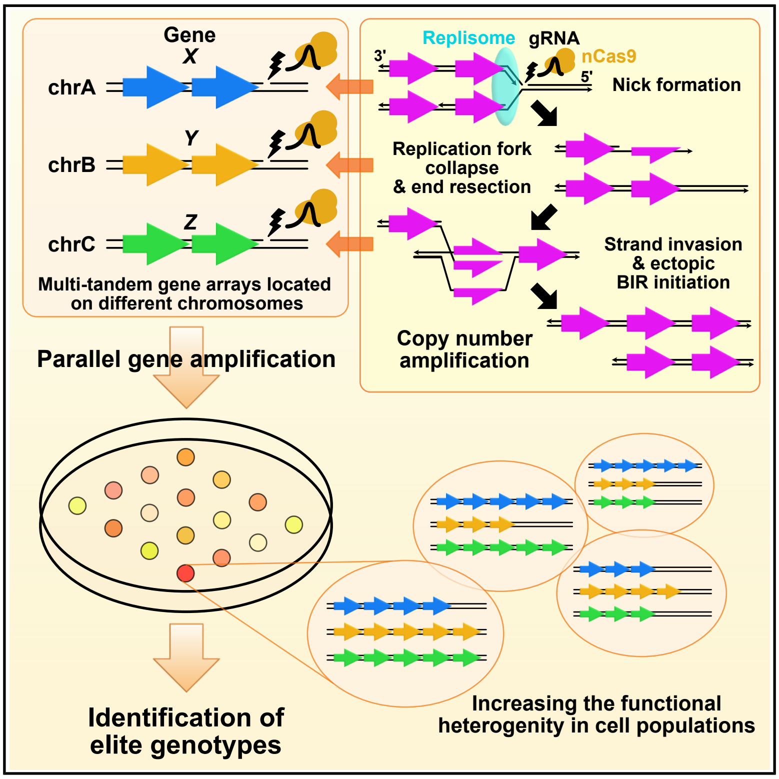

Highlights

- Parallel BITREx diversifies gene copy numbers and stoichiometry in budding yeast
- It creates functionally heterogeneous cell populations for phenotypic screening
- It helps identify elite yeast genotypes for optimized β-carotene production

Authors

Hiroaki Takesue, Satoshi Okada, Takashi Ito

Correspondence

ito.takashi.352@m.kyushu-u.ac.jp

In brief

Takesue et al. present parallel BITREx to randomize the copy numbers and stoichiometry of cooperating genes in yeast. This platform generates cell populations with diverse functional performances by diversifying gene dosage, offering an approach to expanding phenotypic heterogeneity for broad applications in basic and applied research.

Takesue et al., 2026, Cell Reports Methods *6*, 101467
July 20, 2026 © 2026 The Author(s). Published by Elsevier Inc.
https://doi.org/10.1016/j.crmeth.2026.101467

CellPress

Cell Reports Methods

CellPress
OPEN ACCESS

Article

Parallel gene amplification by Cas9 nickase for generating functionally heterogeneous cell populations

Hiroaki Takesue,[1,2] Satoshi Okada,[1,3] and Takashi Ito[1,2,4,*]

[1]Department of Biochemistry, Kyushu University Graduate School of Medical Sciences, Fukuoka 812-8582, Japan
[2]Present address: Division of Transcriptomics, Medical Institute of Bioregulation, Kyushu University, Fukuoka 812-8582, Japan
[3]Present address: Department of Biological Science, Faculty of Science and Engineering, Yasuda Women's University, Hiroshima 731-0153, Japan
[4]Lead contact
*Correspondence: ito.takashi.352@m.kyushu-u.ac.jp
https://doi.org/10.1016/j.crmeth.2026.101467

MOTIVATION Biological functions emerge from the coordinated action of multiple gene products. Consequently, the stoichiometry of these components crucially dictates the performance of molecular pathways. Genetic variation in the dosage of cooperating genes should thus provide the raw material for rapid adaptive evolution and a robust platform for the artificial enhancement of cellular traits. We reasoned that the combinatorial randomization of gene copy numbers would effectively expand this functional potential by exploring a vast dosage space. To realize this goal, we applied our Cas9 nickase-based gene amplification method to generate functionally heterogeneous cell populations, enabling the systematic exploration of optimal gene dosages for enhanced biological functions.

SUMMARY

Genetic diversity underlies adaptive evolution. Because genes often act in concert to execute biological processes, the dosage stoichiometry among cooperating genes represents an additional layer of diversity beyond sequence variation. We therefore hypothesized that combinatorial randomization of gene copy numbers could generate cell populations enriched for functional heterogeneity and evolutionary potential. To test this idea, we extended our previously developed Cas9 nickase-based gene amplification method, break-induced replication-mediated tandem repeat expansion (BITREx), to simultaneously target multiple genes. Applying parallel BITREx to three carotenogenic genes introduced into the budding yeast *Saccharomyces cerevisiae*, we generated a cell population exhibiting broad variation in both absolute copy numbers and their stoichiometric ratios. This population enabled the identification of elite genotypes—specific copy number combinations that conferred enhanced β-carotene production. These results suggest that parallel BITREx is a versatile strategy for increasing functional heterogeneity in cell populations, with potential applications in both basic and applied research.

INTRODUCTION

Genetic diversity within a population is essential for adaptive evolution by natural selection.[1,2] Populations with greater genetic diversity generally exhibit higher evolutionary potential, whereas highly homogeneous populations are more susceptible to extinction. Accordingly, enhancing genetic diversity can improve robustness to environmental changes and increase evolvability.

Genetic diversity influences biological function through both quantitative and qualitative changes in gene activity. Coding variants may reduce activity (hypomorph), enhance activity (hypermorph), or, less frequently, confer novel activities (neomorph). Hypermorphic effects can also arise either from elevated expression by non-coding variants or from dosage effects due to gene amplification.

Gene amplification serves as a powerful means for rapid adaptation and has contributed to the evolution of many adaptive traits. For example, increased amylase gene copy numbers in humans and dogs are thought to reflect their adaptation to starch-rich diets, a change not observed in their sister lineages, chimpanzees and wolves.[3] These variants likely conferred a selective advantage after the advent of agriculture, whereas their counterparts in chimpanzees and wolves offered no such benefit in the wild.[4] Similarly, domesticated strains of the budding yeast

© 2026 The Author(s). Published by Elsevier Inc.
This is an open access article under the CC BY license (http://creativecommons.org/licenses/by/4.0/).

Saccharomyces cerevisiae have adapted to higher copper levels than their wild ancestors by tandemly amplifying the metallothionein gene *CUP1*.[5] Strain-specific repeat unit boundaries indicate that the initial duplications of the single-copy *CUP1* gene occurred independently in different lineages, exemplifying convergent evolution.[6] A strikingly recent example is the adaptation of crop weeds to glyphosate-rich environments—glyphosate having been introduced as a herbicide in the mid-1970s—through amplification of the 5-enolpyruvylshikimate-3-phosphate synthase (*EPSPS*) gene.[7] *EPSPS* amplification has been reported in eight species, with one shown to use tandem duplication and another heritable extrachromosomal circular DNA, illustrating rapid convergent evolution driven by distinct gene amplification mechanisms.[7] In biotechnology, increasing the copy number of biosynthetic genes—whether on episomal plasmids or within host chromosomes—is a widely used strategy to enhance production. This artificial gene amplification can be regarded as a form of adaptation to human-imposed selective pressure.

Importantly, genes rarely act in isolation; rather, they typically function in concert to carry out biological roles. For instance, the efficiency of a metabolic pathway depends on the coordinated action of its constituent enzymes. As a result, pathway performance is determined not only by the catalytic properties of individual enzymes but also by their expression levels and stoichiometric balance. Consequently, variation in the copy number of these genes can modulate pathway output.

To investigate this concept, a method is required to increase the copy number of genes of interest. Because strict copy number control is difficult to achieve with episomal plasmid vectors, amplification of genomic copies represents a more promising strategy. In this context, it should be noted that we and others have developed genome editing approaches for gene duplication using either Cas9 nickase (nCas9)[8,9] or prime editors.[10,11] Among these, our nCas9-based gene amplification strategy, termed break-induced replication (BIR)-mediated tandem repeat expansion (BITREx), is specifically designed to extend tandem gene arrays.[9] BITREx positions nCas9 at a site flanking a target tandem gene array, thereby breaking the replication fork to create a single-ended double-strand break (seDSB) (Figure 1A). The break is end-resected to produce single-stranded DNA (ssDNA), which invades the unbroken sister chromatid to initiate displacement DNA synthesis or BIR. Occasionally, the ssDNA mis-invades an upstream repeat unit, initiating ectopic BIR that expands the tandem array. Our previous work demonstrated that prolonged BITREx treatment can expand the yeast *CUP1* array from 28 kb—corresponding to 14 copies of a 2-kb repeat units—to over 1 Mb, exceeding 500 copies.[9] We also showed that appropriate splint DNAs allow BITREx to generate tandem gene arrays *de novo* from single-copy genes.[9] Furthermore, we demonstrated the applicability of BITREx in mammalian cells.[9]

Here, we applied BITREx simultaneously to two or three gene arrays, generating populations with diverse copy number combinations. By simultaneously increasing the copy numbers of pathway genes and altering their stoichiometric balance, parallel BITREx can generate cell populations that are functionally diversified at the pathway level, thereby enhancing their potential for adaptive evolution.

RESULTS

Rationale of parallel BITREx

BITREx is a replication-coupled process that generates genetic diversity between two daughter cells. It expands the tandem gene array exclusively on the broken chromatid (acceptor) while leaving the intact chromatid (donor) unchanged (Figure 1A). For example, if a single round of BITREx at the S phase adds one repeat unit to a cell carrying a two-unit tandem array, the donor and acceptor chromatids carry two- and three-unit arrays, respectively. During the subsequent M phase, one daughter inherits the unmodified array, whereas the other necessarily inherits the expanded array. Thus, BITREx intrinsically drives copy number divergence between progenies.

Now consider two neighboring genes, X and Y, forming a $2\times(X+Y)$ array (Figure 1B). If BITREx expands this array by one $X+Y$ repeat unit each cell cycle, then the population after two generations will contain cells with total $X+Y$ copy numbers ranging from two to four, or three genotypes. Crucially, however, the dosage ratio of X to Y remains locked at one (i.e., 2:2, 3:3, or 4:4), irrespective of the total copy number. In other words, tandem BITREx increases variation in target gene copy numbers but not in their stoichiometric ratios.

Next, consider parallel BITREx applied to $2\times X$ and $2\times Y$ arrays located on different chromosomes (Figure 1C). Because BITREx produces one expanded allele and one unaltered allele of each array, they are transmitted to daughter cells in two distinct ways, co-segregation or separation. In co-segregation, one daughter cell inherits both expanded alleles ($3\times X$ and $3\times Y$), while the other necessarily receives the original alleles ($2\times X$ and $2\times Y$). In separation, one daughter inherits $2\times X$ and $3\times Y$, while the other inherits $3\times X$ and $2\times Y$. Over successive cell cycles, each lineage can continue to diversify through either mode, gradually producing a spectrum of copy number combinations that deviate from the original stoichiometric ratio of one. Therefore, parallel BITREx increases variation not only in copy numbers but also in their stoichiometric ratios.

At the population level, copy number combinations, or genotypes, increase with each generation. Beginning with two copies of X and Y, copy numbers after N generations can increase to as many as $N + 2$, producing $N + 1$ distinct alleles per locus. Because X and Y are inherited independently, the total number of possible genotypes becomes $(N + 1)^2$. More generally, parallel BITREx applied to m genes can generate $(N + 1)^m$ genotypes in the population after N generations. This simple model highlights the combinatorial power of parallel BITREx to rapidly generate genetic heterogeneity within a population.

Diversification of target gene copy numbers by parallel BITREx

To examine the feasibility of parallel BITREx, we first asked whether simultaneous induction of BITREx at two genomic loci would interfere with one another and thereby compromise the expansion efficiency of each target array.

For this purpose, we employed the estradiol-inducible BITREx system in the budding yeast *S. cerevisiae* established in our previous study[9] (Figure 2A). This system employs the synthetic estrogen-responsive transcription factor GEV, consisting of the

CellPress
OPEN ACCESS

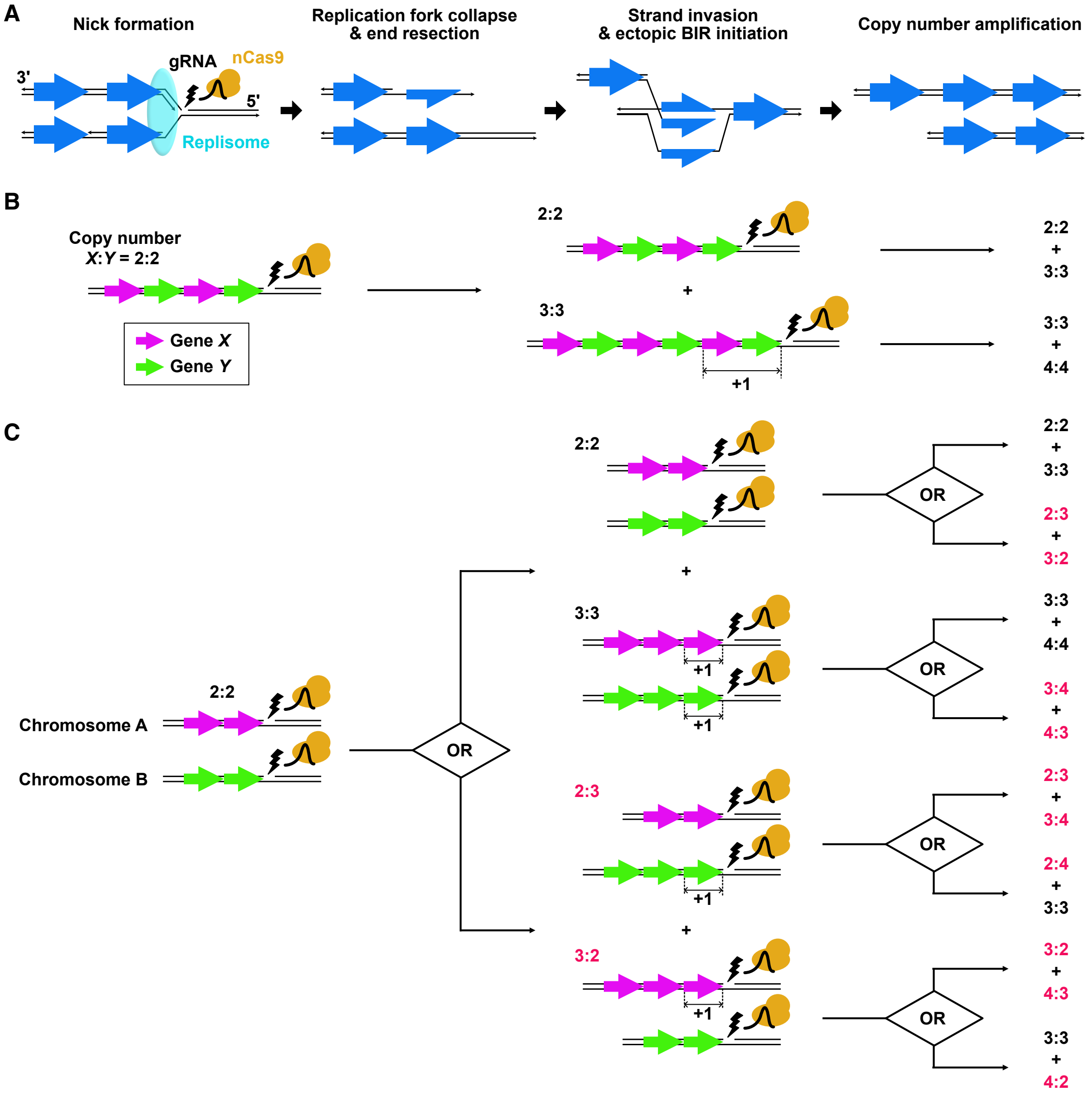

Figure 1. Rationale of parallel BITREx

(A) Principle of BITREx. After replication of the target gene array (depicted as two blue arrows), the replisome encounters an nCas9-induced nick in the flanking region, leading to replication fork collapse and seDSB formation. The seDSB undergoes end resection to produce ssDNA, which invades the unbroken sister chromatid to initiate BIR. Occasionally, the ssDNA mis-invades an upstream repeat unit, triggering ectopic BIR that expands the array on the broken chromatid but not on the intact one. For simplicity, a two-unit array is shown as the target, and a single round of BITREx is illustrated to add one repeat unit. Note that the two daughter cells inheriting the individual chromatids differ in their repeat unit numbers.

(B) Single-locus tandem BITREx. When the repeat unit of a BITREx target consists of two genes, *X* and *Y* (i.e., a $2\times(X+Y)$ array), both genes are amplified simultaneously. Although cells in later populations exhibit a range of absolute copy numbers, the stoichiometric ratio of *X* to *Y* remains fixed at one. The trajectory of the copy number ratio is shown for two consecutive BITREx cycles, leading to the generation of three genotypes.

(C) Multi-locus parallel BITREx. When two BITREx targets are located on different chromosomes as $2\times X$ and $2\times Y$ arrays, the expanded arrays may either co-segregate or separate during the mitosis after BITREx. This generates progenies with stoichiometric ratios deviating from the original value of one, with altered ratios highlighted in red. The trajectory of the copy number ratio is shown for two consecutive BITREx cycles, leading to the generation of nine genotypes.

yeast Gal4 DNA-binding domain (G), the human estrogen receptor α ligand-binding domain (E), and the herpes simplex virus VP16 transactivator domain (V). Upon addition of β-estradiol, GEV binds the ligand, translocates into the nucleus, and drives expression of both nCas9 mRNA from the genome and the guide RNA (gRNA) from a plasmid, both under the control of the *GAL1* promoter. The gRNA is flanked by hammerhead and hepatitis delta virus (HDV) ribozymes, which self-cleave to release the gRNA from the primary transcript synthesized by RNA polymerase II.

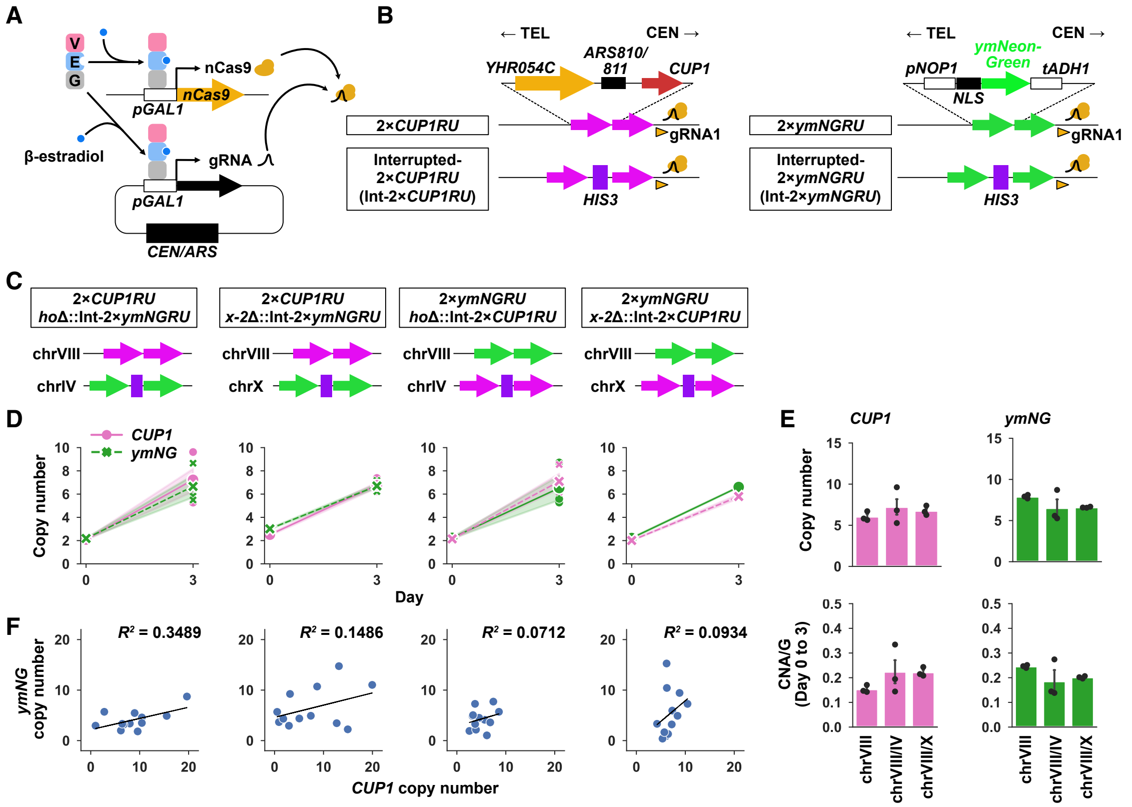

Figure 2. Proof of principle for parallel BITREx

(A) GEV-based system for co-induction of nCas9 and gRNA. G, yeast Gal4 DNA-binding domain; E, human estrogen receptor α ligand-binding domain; V, the herpes simplex virus VP16 transactivator domain.

(B) Four two-unit arrays used for this series of experiments. Magenta and green arrows indicate *CUP1RU* and *ymNGRU*, respectively, and were used to generate respective two-unit arrays in both uninterrupted and *HIS3*-interrupted forms.

(C) Four strains bearing two target gene arrays. The 2×*CUP1RU* and 2×*ymNGRU* arrays were integrated into the *CUP1* locus on chromosome VIII. Their *HIS3*-interrupted versions were integrated at the *HO* locus on chromosome IV or the *X-2* locus on chromosome X.

(D) Copy number alterations of *CUP1* and *ymNG* genes. Results for the four strains shown in (C) are presented below the corresponding strains. Copy numbers were quantified by qPCR at days 0 and 3 of BITREx. Data are represented as mean ± standard deviation (SD) (n = 3 biological replicates). Each point in the line plot represents the mean copy number, with the shaded area around each line indicating the SD.

(E) Efficiency of BITREx in the absence and presence of an additional target array. The upper images show copy numbers at day 3, whereas the lower images indicate copy number alteration per generation (CNA/G). Because copy number increases in only one of the two daughter cells, the 2×CNA/G value serves as a proxy for the number of repeat units increased by a single round of BITREx. Data are represented as mean ± SD (n = 3 biological replicates).

(F) Copy number distribution after 3-day BITREx. Results for the four strains shown in (C) are presented below each corresponding strain. Copy numbers of *CUP1* and *ymNG* were quantified in 12 randomly selected clones per strain.

As targets for parallel BITREx, we designed four two-unit gene arrays (Figure 2B). Each array either contained or lacked the *HIS3* gene between the two repeat units, but all shared a common flanking sequence recognized by gRNA1-directed nCas9. The repeat units were: (1) the *CUP1* repeat unit (CUP1RU), composed of the *CUP1* gene, the replication origin *ARS810/811*, and the open reading frame *YHR054C*; and (2) the yeast codon-optimized monomeric NeonGreen repeat unit (ymNGRU), composed of a *ymNG* gene preceded by the promoter and nuclear localization signal (NLS)-coding sequence of *NOP1* and followed by the *ADH1* terminator (*tADH1*). These arrays were integrated at the *CUP1* locus on chromosome VIII and at either the *HO* locus on chromosome IV or the *X-2* safe harbor locus on chromosome X,[12] generating four distinct strains (Figure 2C).

We induced BITREx in these strains with β-estradiol and quantified *CUP1* and *ymNG* copy numbers by quantitative PCR (qPCR) at days 0 and 3. Both arrays expanded in all four strains (Figure 2D). Using qPCR and cell proliferation data, we calculated the copy number alteration per generation (CNA/G) for the 2×*CUP1RU* and 2×*ymNGRU* arrays on chromosome VIII. We then compared these values between strains with or without an additional target array on chromosome IV or X (Figure 2E). CNA/G values were comparable regardless of the presence or absence of a second target, suggesting that BITREx events at different chromosomes do not interfere with one another but rather proceed independently.

Next, we isolated 12 clones from each population on day 3 and measured *CUP1* and *ymNG* copy numbers (Figure 2F). Diverse

copy number combinations (genotypes) were observed even in this small sampling size, demonstrating that parallel BITREx can rapidly generate genetic diversity.

Parallel BITREx in diploid cells

We next investigated whether parallel BITREx remains effective in diploid cells, which possess twice as many BITREx-compatible sites and could thereby increase the number of target genes per cell (Figure 3A). To this end, we first constructed four **a**-type and four α-type haploid strains, each carrying either the 2×*CUP1RU* or 2×*ymNGRU* array at the *CUP1* locus on chromosome VIII, or their *HIS*3-interrupted variant at the *X-2* locus on chromosome X. By crossing these haploids, we generated eight diploid strains, each harboring two target arrays in either a heterozygous or hemizygous configuration.

In the heterozygous strains, the two arrays were inserted at allelic positions. Strains #1 and #2 were constructed by reciprocal crosses of the parental haploids to carry the 2×*CUP1RU* and 2×*ymNGRU* arrays at the *CUP1* loci on the two copies of chromosome VIII. Likewise, strains #3 and #4 carried the *HIS3*-interrupted variants of 2×*CUP1RU* and 2×*ymNGRU* arrays at the *X-2* loci on the two copies of chromosome X (Figure 3A).

In the hemizygous strains, the two arrays were inserted at nonallelic positions. Strains #5 and #6, generated by reciprocal crosses of the parental haploids, carried the 2×*CUP1RU* array at the *CUP1* locus and the *HIS3*-interrupted 2×*ymNGRU* array at the *X-2* locus, with the alternate alleles remaining null (i.e., lacking inserted gene arrays). Conversely, strains #7 and #8 carried the 2×*ymNGRU* array at the *CUP1* locus and the *HIS3*-interrupted 2×*CUP1RU* array at the *X-2* locus, again with null alternate alleles (Figure 3A).

Upon BITREx induction, both arrays expanded in all eight diploid strains (Figure 3B). We next compared the extent of expansion in diploid cells with that observed in their parental haploid strains (Figure 3C). In most cases, all four array types expanded to comparable levels in diploids and haploids. Notably, however, certain arrays exhibited even greater expansion. For example, the 2×*CUP1RU* array derived from the **a**-type haploid showed significantly higher CNA/G in hemizygous diploid strain #5. Likewise, the *HIS3*-interrupted versions of 2×*CUP1RU* array derived from **a**-type haploid expanded to a greater extent in the corresponding heterozygous diploid strain #3. It is formally possible that the observed bias results from loss of heterozygosity induced by inter-homolog or inter-chromosomal BIR, particularly given the presence of a single *CUP1RU* left on the chromosome VIII inherited from the α-type haploid (see Figure 3A legend) and the *HIS3* marker interrupting both two-unit arrays. However, our nanopore sequencing analysis failed to find evidence for such events occurring at a frequency sufficient to impact the population-averaged increase in *CUP1* copy numbers. Additionally, the 2×*ymNGRU* array exhibited different efficiencies between **a**-type and α-type haploids ($p < 0.001$), and this difference was faithfully transmitted to the diploids derived from them. While a mutation near the nicking site could theoretically affect BITREx efficiency in a heritable manner, our sequencing data revealed no such mutations. Taken together, the causes of these variations remain to be elucidated.

To examine these outcomes in greater detail, we isolated 12 clones per strain on day 3 and quantified *CUP1* and *ymNG* copy numbers (Figure 3D). As before, diverse copy number combinations were observed. Collectively, these results demonstrate that parallel BITREx is also applicable to diploid strains. However, because certain arrays exhibited unexpected behaviors across **a**-type and α-type haploids as well as diploids, it is important to carefully assess BITREx efficiencies.

Parallel BITREx for improved β-carotene synthesis in baker's yeast

Having proved the concept of parallel BITREx, we next asked whether randomizing the copy numbers of cooperating genes could indeed enhance functional heterogeneity within a cell population.

As a model to address this question, we turned to engineering *S. cerevisiae* for β-carotene production. Heterologous β-carotene production in safe hosts, such as baker's yeast, has drawn considerable interest, as β-carotene serves not only as a widely used food pigment but also as a vitamin A precursor with potential antioxidant benefits in humans. The introduction of three carotenogenic genes—*crtE*, *crtI*, and *crtYB* from the red yeast *Xanthophyllomyces dendrorhous*—has been shown to enable *S. cerevisiae* to synthesize β-carotene.[13] Together, the enzymes encoded by these genes redirect farnesyl pyrophosphate, normally used by *S. cerevisiae* for the synthesis of sterols, dolichols, ubiquinone, certain hemes, and for protein prenylation, toward β-carotene production. Because overexpression of these genes from an episomal vector leads to strain instability, recent studies have instead integrated them into the yeast genome.[13] We therefore hypothesized that randomizing their copy numbers via parallel BITREx would generate a cell population with heterogeneous capacities for β-carotene production.

To render each gene amenable to BITREx, we placed it between two *LEU2* terminators (*tLEU2*) to create a 2×*tLEU2* array interrupted by the respective carotenogenic gene (Figure 4A). These configurations are analogous to the *HIS3*-interrupted 2×*CUP1RU* and 2×*ymNGRU* arrays used above. Each carotenogenic gene is placed under the control of a Tet-On promoter (*P7tet.1*) for doxycycline (Dox)-induced activation (derepression)[14] and is flanked by *ARS305* to enable continuous BITREx, as demonstrated in our previous study.[9] The 2×*tLEU2* arrays interrupted by *crtE*, *crtI*, and *crtYB* were integrated into the *XI-1* locus on chromosome XI,[15] the *X-2* locus on chromosome X,[12] and the *CUP1* locus on chromosome VIII, respectively (Figure 4A). Each integration included a distinct selection marker gene and an nCas9 target site (Figure 4A).

We selected two clones from the strain with the three integrated genes and cultured them in the presence of β-estradiol to induce parallel BITREx (Figure 4B). At the outset, *crtI* and *crtYB* were present as single copies, whereas *crtE* unexpectedly had four copies. This initial asymmetry likely arose from stochastic multiple tandem integration of the transforming DNA during strain construction, as has been previously documented.[16,17] After 3 days of parallel BITREx, the average copy numbers of all three carotenogenic genes increased in both clones. Clone #1 was chosen for further study because it exhibited stronger *crtI* amplification than clone #2.

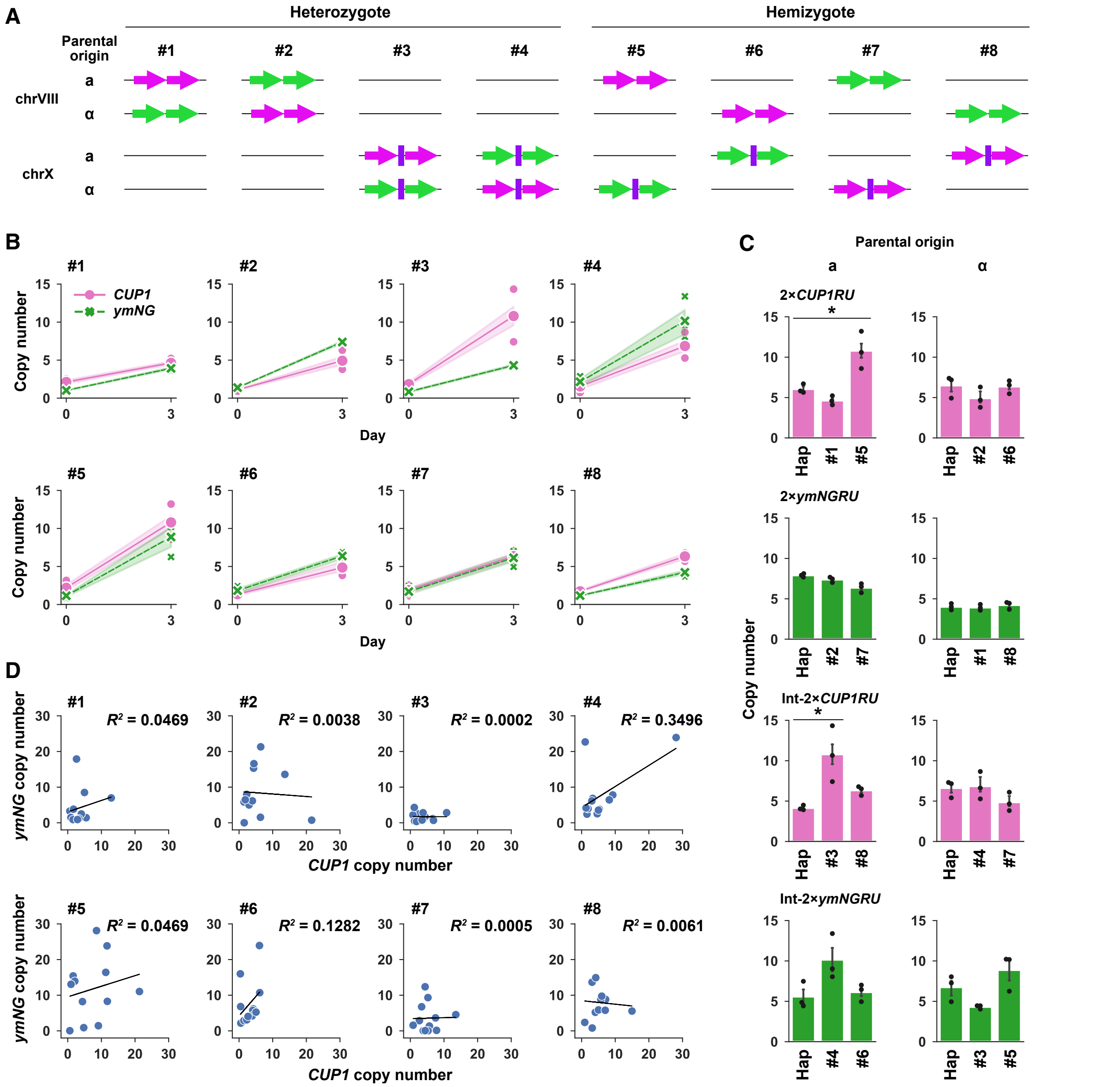

Figure 3. Parallel BITREx in diploid cells

(A) Eight diploid strains bearing two target arrays. The 2×*CUP1RU* and 2×*ymNGRU* arrays were integrated into the *CUP1* locus on chromosome VIII, while their *HIS3*-interrupted versions were integrated at the *X-2* locus on chromosome X. Strains #1–#4 and #5–#8 are heterozygous and hemizygous, respectively, for the integrated arrays. The four lines in each strain indicate the chromosomes VIII and X derived from its **a**-type and α-type parental haploid strains. The original *CUP1* array composed of ∼14 copies of *CUP1RU* was fully replaced by the *NatMX* cassette in the **a**-type haploid strain, whereas it was contracted to a single copy of *CUP1RU* in the α-type haploid strain (Table S1). Since neither the *NatMX* cassette nor the single *CUP1RU* can be expanded by inter-chromatid BIR, they are omitted from the schematic for visual simplicity.

(B) Copy number alterations of *CUP1* and *ymNG* genes in the eight diploid strains shown in (A). Copy numbers were quantified by qPCR at days 0 and 3 of BITREx. Data are represented as mean ± SD ($n = 3$ biological replicates). Each point in the line plot represents the mean copy number, with the shaded area around each line indicating the SD.

(C) Comparison of BITREx efficiency between parental haploid and corresponding diploid strains. Copy numbers of each target array after 3-day BITREx were compared between the parental haploid strains and their corresponding diploid strains. Data are represented as mean ± SD ($n = 3$ biological replicates). *$p < 0.05$ (Student's t test). "Hap" denotes haploid strain.

(D) Copy number distribution after 3-day BITREx. Copy numbers of *CUP1* and *ymNG* were quantified in 12 randomly selected clones per strain.

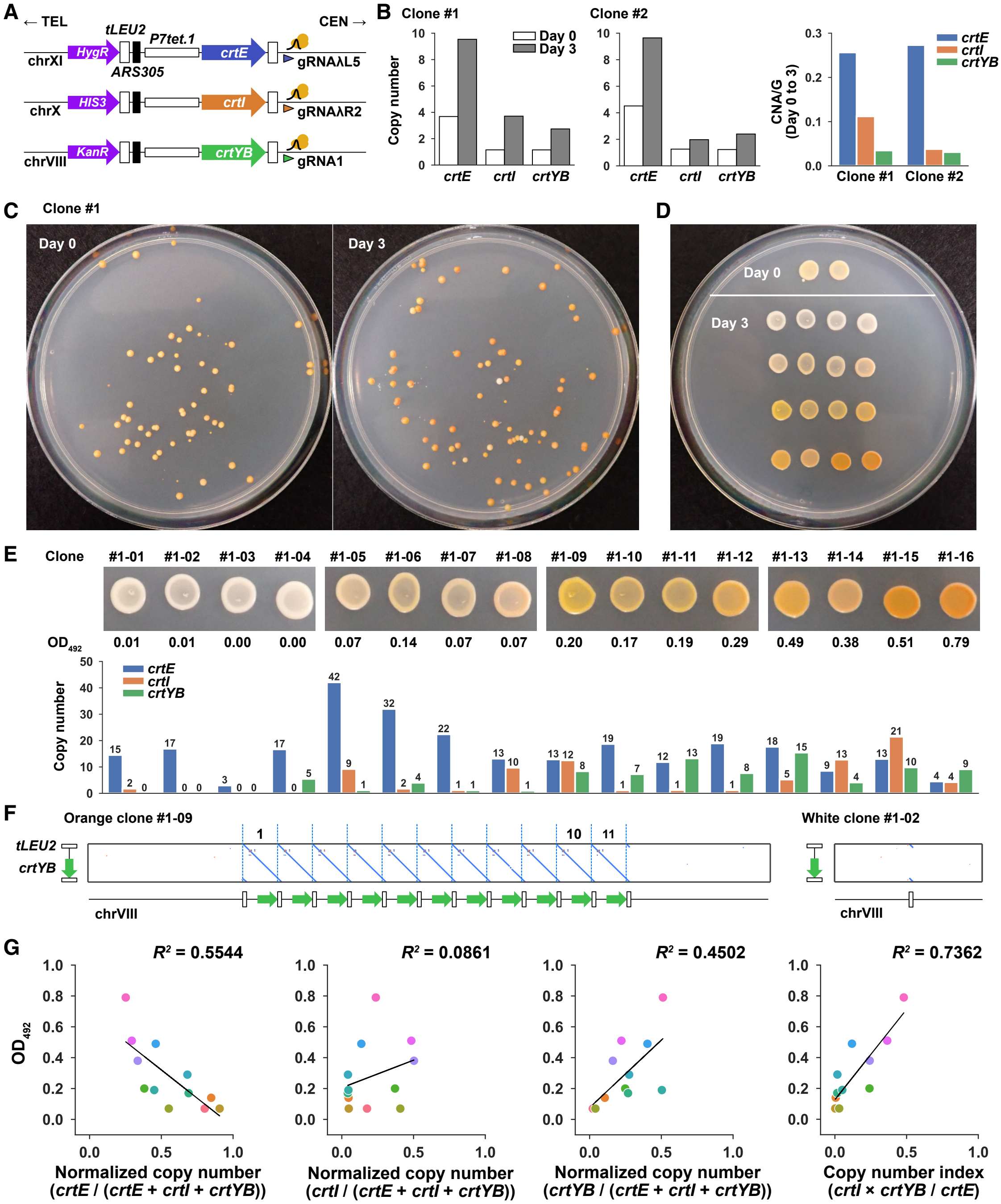

Figure 4. Phenotypic variation generated by parallel BITREx of three carotenogenic genes

(A) Target genes for parallel BITREx. Each gene involved in β-carotene biosynthesis was integrated to interrupt 2×*tLEU2* array to be amplified by BITREx. Each carotenogenic gene was under the control of the Dox-inducible *P7tet.1* promoter and flanked by *ARS305*, allowing expansion to continue. Selection markers used for strain construction and gRNA target sites are also indicated.

(B) Copy number alterations of the three carotenogenic genes. Two randomly selected clones #1 and #2 were subjected to BITREx and numbers of each target gene at days 0 and 3 were quantified by qPCR (left and middle images). CNA/G values were also calculated (right).

(C) Colonies on Dox-containing agar plates to induce expression of the three carotenogenic genes. Left and right plates show colonies of clone #1 at day 0 and day 3 of BITREx, respectively.

(D) Spots of 16 clones selected for copy number analysis in (E). Four representative clones of each colony-color class were chosen from the day-3 plate in (C). For comparison, two clones from the day-0 plate in (C) were also included.

(legend continued on next page)

We next plated clone #1 cells from day 0 and day 3 cultures on agar plates containing Dox to induce expression of the three carotenogenic genes (Figure 4C). Colonies derived from the day 0 culture, with the initial copy numbers, consistently appeared faintly orange. By contrast, colonies from the day 3 culture exhibited a broader spectrum of orange intensities: most were more deeply orange than those from the day 0 culture, whereas a subset appeared white.

To explore this variability, we isolated clones displaying different colony colors (Figure 4D) and determined their carotenogenic gene copy numbers by nanopore sequencing (Figure 4E). Copy numbers were estimated by counting the occurrences of each gene sequence in the reads and normalizing them to the genome-wide average read depth. As expected, clones with different shades of orange carried distinct combinations of gene copies. To gain structural insight into these differences, we selected reads spanning the target gene arrays (i.e., reads containing both upstream and downstream flanking sequences) and compared them with the reference sequences of the arrays using dot plots (Figure 4F). These plots unequivocally demonstrate that the observed copy number increases resulted from the expansion of the target gene arrays. In addition, dot plots of reads from white clones, which appeared at a frequency of ∼2.5% (4 in 158 colonies), revealed deletions of either *crtI* or *crtYB*, leaving a single *tLEU2* behind (Figure 4F). Such deletions are most likely caused by extensive end resection reaching into the upstream *tLEU2*, which then invaded the downstream *tLEU2* on the donor chromatid to initiate ectopic BIR leading to array contraction, as observed and discussed in our previous study.[9]

The copy numbers of the three carotenogenic genes did not provide a straightforward explanation for the observed colony colors. To assess the contribution of each gene, we correlated its copy number with β-carotene levels, as determined by the absorbance of cell extracts at 492 nm, which serves as a high-throughput proxy for the total pigment titer (Figure 4G). For this analysis, we employed normalized copy numbers, defined as the dosage of each gene relative to the total copy number of the three pathway genes, rather than raw copy numbers. This normalization was necessitated by the architecture of our system, in which all three genes are driven by the same *P7tet.1* promoter (a Dox-derepressible derivative of the strong *TDH3* promoter).[14] We reasoned that as the total copy number increases, these identical promoters could potentially compete for a limited pool of endogenous transcription factors that activate the *TDH3* promoter, thereby modulating the effective transcriptional output per gene. The analysis indicated only a slight positive trend for *crtYB*, no apparent relationship for *crtI*, and a slight negative trend for *crtE*. Intriguingly, a combined index calculated from the three normalized copy numbers showed a stronger correlation with the carotenoid yield (R^2 = 0.7362; Figure 4G).

We next sought to independently validate the identified elite genotypes by attempting to recapitulate the copy number stoichiometry of clone #1-16 starting from a distinct parental strain. This alternative strain had already acquired four copies each of *crtE* and *crtI* at the initial strain construction phase (Figure S1A). We therefore applied simplex BITREx to specifically expand the *crtYB* copy number in this strain (Figure S1B). Although we did not isolate clones with an identical copy number combination, those with approximate stoichiometries consistently exhibited high levels of β-carotene production (Figures S1C and S1D). While this experiment still utilizes the BITREx system and thus does not constitute a strictly orthogonal validation, it demonstrates the reproducibility of the elite phenotype. These findings align with the elite genotypes identified in the initial parallel BITREx screen and provide supportive evidence for the functional relevance of the optimized gene dosage.

The stability of elite genotypes is a key consideration for the practical application of parallel BITREx. To evaluate this, we monitored copy-number retention via qPCR over ∼30 generations (4 days) of passaging in the absence of β-estradiol (i.e., without BITREx induction; Figure S2A). The results demonstrate that copy numbers remained largely comparable between day 0 and day 4; notably, the top four elite clones consistently maintained their superior positions in terms of production phenotypes (Figure S2B), even if minor alterations in copy number occurred. On the other hand, the degree of stability exhibited variability among the clones examined. For instance, while clone #1-5 stably maintained 45 copies of *crtE*, clone #1-6 showed a more pronounced contraction of the *crtE* array from 32 to 20 copies (a 37% reduction) (Figure S2A). This indicates that even the similar gene arrays integrated at the same locus can exhibit differential stability across independent clones. Therefore, although most clones show sufficient stability for standard laboratory applications, we recommend rigorous verification of copy-number retention when maintaining elite clones for long-term or large-scale industrial applications, as the inherent instability of high-copy tandem repeats cannot be entirely ruled out.

DISCUSSION

In this study, we introduced the concept of parallel BITREx as a strategy for combinatorial randomization of gene copy numbers, thereby creating cell populations endowed with high functional diversity. Such populations represent not only a valuable resource for applied strain engineering but also a platform for probing evolutionary principles, since they harbor broad phenotypic space accessible to selection. Notably, because BITREx inherently produces asymmetric copy number outcomes in daughter cells, its parallel version multiplies heterogeneity across a population, as predicted by our simple modeling (Figure 1).

(E) Copy numbers of the three carotenogenic genes in the 16 clones in (D). The top image shows each clone patch alongside the OD_{492} value of the cell extract, serving as a proxy for β-carotene yield. The bar graph indicates copy numbers estimated from nanopore sequencing data.

(F) Representative dot plots comparing nanopore reads to the reference sequence. Reads from orange and white clones were compared to the reference sequence of the *crtYB*-interrupted 2×*tLEU2* array integrated at the *CUP1* locus on chromosome VIII.

(G) Correlation between normalized gene copy numbers and β-carotene yield. The normalized gene copy number is defined as its individual gene copy number relative to the total copy number of the three genes in the cell. This analysis includes the 12 orange clones (#1-05 to #1-16) identified in (D). The rightmost image shows the correlation between β-carotene yield and a combined index calculated from the normalized copy numbers.

CellPress
OPEN ACCESS

We should note that the model was overly simplified, and actual parallel BITREx is likely to be even more unpredictable. This is partly because, even when nCas9-mediated replication fork collapse occurs with 100% efficiency, ectopic BIR events that drive BITREx compete with orthotopic BIR events that correctly repair the array on the broken chromatid. Moreover, the number of repeat units added during each BITREx cycle is not constant but variable. Extensive end resection can also lead to array contraction during BITREx, particularly when the array is relatively short, as exemplified in our previous study.[9] This problem is likely mitigated by limiting the extent of end resection. In this context, it is noteworthy that the catalytically inactive Cas9 variant (dCas9) can attenuate DSB end resection.[18] While this strategy may be effective in suppressing contraction of short arrays, targeting dCas9 to long arrays is likely to accelerate their contraction instead.[19]

Using four model two-unit arrays in baker's yeast, we demonstrated that parallel BITREx maintains the efficiency of single-array expansion while amplifying diversity in array length across the population (Figures 2 and 3). When applied to a heterologous β-carotene synthesis pathway, parallel BITREx generated a population with diverse absolute copy numbers and stoichiometric ratios of the three carotenogenic genes (Figure 4). Importantly, this population yielded colonies with widely variable β-carotene outputs, including elite clones with substantially improved production (Figures 4 and S1).

The genotypes underlying these enhanced phenotypes were neither intuitive nor readily predictable, underscoring the difficulty of rationally designing optimal copy number combinations. Moreover, the optimal dosage ratio is likely dependent on the specific genetic architecture used to express the genes. For instance, a previous study reported an effect of increased *crtI* dosage,[13] whereas a more recent study emphasized the role of *crtYB*.[20] Together, these findings suggest a complex, genetic context-dependent interplay among the three carotenogenic genes expressed in *S. cerevisiae*, making it challenging to predict optimal copy number configurations *a priori*. Consequently, identifying the most productive genotypes for a given specific context is more effectively achieved empirically through parallel BITREx.

Parallel BITREx requires at least two genomic loci competent for BITREx. Our previous work revealed differential competence among loci: identical arrays integrated at different sites sometimes exhibited markedly different efficiencies.[9] In this study, we used four validated loci; however, expanding the repertoire of BITREx-competent loci would further enhance the flexibility and potential of this approach. Moreover, to fully exploit parallel BITREx, these loci must reside on different chromosomes to ensure independent segregation of donor and acceptor arrays at each locus. Consequently, the number of chromosomes limits the number of ideal BITREx targets.

To address these issues, we demonstrated the feasibility of parallel BITREx in diploid cells by exploiting heterozygosity at a single BITREx-competent locus to simultaneously expand two distinct gene arrays (Figure 3). This heterozygous BITREx would not only circumvent the need to search for new BITREx-competent loci but also facilitate the application of parallel BITREx to organisms with few chromosomes, such as the fission yeast *Schizosaccharomyces pombe*, which carries three chromosomes.

The phenotypic diversity achieved by parallel BITREx could, in principle, be pursued through conventional combinatorial promoter engineering. For example, a previous study utilized a set of five LexA-driven promoters with varying strengths—conferred by 0, 1, 2, 4, or 8 LexA-binding sites—to randomly replace three target gene promoters by homology-dependent recombination-based genome editing using Cas9.[21] This approach theoretically yields a discrete phenotypic space of 125 (=5^3) potential combinations. While such promoter shuffling methods can be applied to a larger number of genes (unconstrained by chromosome number) and may yield more genetically stable strains than those generated by BITREx, they are often limited in practice by the scarcity of distinct, well-characterized promoters. Furthermore, the maximum expression level in these systems is inherently capped by the strongest available promoter in the set.

In contrast, the gene copy numbers achievable by BITREx are not strictly limited, allowing for the exploration of a much larger gene dosage space. By sampling clones at multiple time points during long-term BITREx induction, a significantly wider range of copy numbers can be accessed than through the single-point sampling employed in this study. Critically, by multiplying a gene even under the control of a maximally strong promoter, BITREx can achieve expression levels previously unattainable through single-copy engineering, provided the overexpression does not reach a threshold of physiological toxicity. These features enable a larger gene dosage space and far less discrete sampling within that space compared to conventional promoter shuffling approaches. Therefore, we believe that parallel BITREx, upon its optimization, provides the capability not only to access the phenotypic space covered by conventional methods but also to significantly expand it, enabling the identification of elite genotypes that otherwise remain unreachable.

It should be noted, however, that the initial copy-number profile of a starter clone—such as the asymmetry observed among the three carotenogenic genes in this study—implicitly defines the lower boundary of the accessible genotype space. Because BITREx predominantly drives gene expansion rather than reduction, any combinations requiring copy numbers lower than the initial state remain inherently underexplored. Consequently, the selection of a starter clone dictates the searchable landscape; to ensure the broadest possible exploration of a fitness landscape, it is generally advisable to initiate the process with the lowest possible copy numbers for all target genes.

While the elite strains identified by parallel BITREx may be used directly for production, careful monitoring of copy number retention is essential to mitigate risks associated with the intrinsic instability of tandemly iterated structures (Figure S2). Alternatively, the expression ratios identified in these elite strains can serve as quantitative blueprint for engineering stable production strains. Such strains could recapitulate the optimal stoichiometry through promoter engineering of single-copy genes. If a single-copy configuration fails to achieve the required expression levels, distributed integration of expression cassettes into multiple genomic loci may provide a viable alternative for maintaining high output with superior genetic stability. Furthermore, the rapid generation of diverse gene-dosage landscapes by parallel BITREx could accelerate the acquisition of high-quality datasets for training predictive models, potentially surpassing the

performance of a recently reported one.[20] These considerations underscore the utility of parallel BITREx as a platform for identifying the optimal transcriptional landscape required for maximum metabolic flux, serving as a critical discovery step prior to final strain optimization.

In summary, we demonstrate the concept of parallel BITREx as a versatile platform for generating functionally heterogeneous cell populations through combinatorial randomization of copy numbers of cooperating genes. Beyond its application in yeast metabolic engineering, as exemplified in this study, this strategy is anticipated, in its optimized forms, to have broad utility in both fundamental research and practical contexts where diversity is essential.

Limitations of the study

First, highly expanded tandem arrays are intrinsically unstable, necessitating close monitoring to sustain desirable traits. Consequently, robust strategies will be required to stabilize or maintain high copy numbers. Alternatively, comparable expression levels to those observed in elite strains should be achieved in strains carrying the genes as single copies by employing an appropriate set of promoters. In addition, multiple seDSBs generated during highly parallel BITREx may impair cell cycle progression and restrict proliferation, thereby constraining the practically achievable degree of parallelism. Finally, the applicability of parallel BITREx to organisms beyond baker's yeast remains to be evaluated.

RESOURCE AVAILABILITY

Lead contact

Further information and requests for resources and reagents should be addressed to and will be fulfilled by the lead contact, Takashi Ito (ito.takashi.352@m.kyushu-u.ac.jp).

Materials availability

Requests for the generated plasmids and strains in this study should be directed to the lead contact, Takashi Ito (ito.takashi.352@m.kyushu-u.ac.jp).

Data and code availability

- All raw sequencing data used in this study were deposited in the DDBJ BioProject database: PRJDB37536, PRJDB40548.
- All original codes used in this study are available from Zenodo at https://doi.org/10.5281/zenodo.11515696.
- Any additional information required to reanalyze the data reported in this work paper is available from the lead contact upon request.

ACKNOWLEDGMENTS

We thank the technical support from the Research Support Center of the Research Center for Human Disease Modeling at Kyushu University Graduate School of Medical Sciences, which is partially supported by the Mitsuaki Shiraishi Fund for Basic Medical Research. This work was supported by JST CREST grant number JPMJCR19S1 and JSPS KAKENHI grant number JP24K02015.

AUTHOR CONTRIBUTIONS

Conceptualization, data curation, resources, and writing – review and editing, H.T., S.O., and T.I.; formal analysis and writing – original draft, H.T.; funding acquisition, project administration, and supervision, T.I.; investigation, methodology, software, validation, and visualization, H.T. and S.O.

DECLARATION OF INTERESTS

The authors declare no competing interests.

DECLARATION OF GENERATIVE AI AND AI-ASSISTED TECHNOLOGIES IN THE WRITING PROCESS

During the preparation of this work, the authors used ChatGPT and Gemini to improve readability of some sentences. After using this tool or service, the authors reviewed and edited the content as needed and take full responsibility for the content of the publication.

STAR★METHODS

Detailed methods are provided in the online version of this paper and include the following:

- KEY RESOURCES TABLE
- EXPERIMENTAL MODEL AND STUDY PARTICIPANT DETAILS
- METHOD DETAILS
  - Yeast strain construction
  - Induction of BITREx
  - Quantitative PCR
  - Nanopore sequencing and data analysis
  - Extraction of β-carotene
- QUANTIFICATION AND STATISTICAL ANALYSIS

SUPPLEMENTAL INFORMATION

Supplemental information can be found online at https://doi.org/10.1016/j.crmeth.2026.101467.

Received: September 30, 2025
Revised: March 31, 2026
Accepted: April 29, 2026
Published: May 25, 2026

REFERENCES

1. Bomblies, K., and Peichel, C.L. (2022). Genetics of adaptation. Proc. Natl. Acad. Sci. USA *119*, e2122152119. https://doi.org/10.1073/pnas.2122152119.
2. Agashe, D., Sane, M., and Singhal, S. (2023). Revisiting the Role of Genetic Variation in Adaptation. Am. Nat. *202*, 486–502. https://doi.org/10.1086/726012.
3. Silverman, S., and Massilani, D. (2025). Double or nothing: Ancient duplications in the amylase locus drove human adaptation. Cell Genom. *5*, 100741. https://doi.org/10.1016/j.xgen.2024.100741.
4. Arendt, M., Cairns, K.M., Ballard, J.W.O., Savolainen, P., and Axelsson, E. (2016). Diet adaptation in dog reflects spread of prehistoric agriculture. Heredity *117*, 301–306. https://doi.org/10.1038/hdy.2016.48.
5. Warringer, J., Zörgö, E., Cubillos, F.A., Zia, A., Gjuvsland, A., Simpson, J. T., Forsmark, A., Durbin, R., Omholt, S.W., Louis, E.J., et al. (2011). Trait variation in yeast is defined by population history. PLoS Genet. *7*, e1002111. https://doi.org/10.1371/journal.pgen.1002111.
6. Zhao, Y., Strope, P.K., Kozmin, S.G., McCusker, J.H., Dietrich, F.S., Kokoska, R.J., and Petes, T.D. (2014). Structures of Naturally Evolved *CUP1* Tandem Arrays in Yeast Indicate That These Arrays Are Generated by Unequal Nonhomologous Recombination. G3 (Bethesda) *4*, 2259–2269. https://doi.org/10.1534/g3.114.012922.
7. Gaines, T.A., Patterson, E.L., and Neve, P. (2019). Molecular mechanisms of adaptive evolution revealed by global selection for glyphosate resistance. New Phytol. *223*, 1770–1775. https://doi.org/10.1111/nph.15858.

8. Sugiyama, Y., Okada, S., Daigaku, Y., Kusumoto, E., and Ito, T. (2024). Strategic targeting of Cas9 nickase induces large segmental duplications. Cell Genom. *4*, 100610. https://doi.org/10.1016/j.xgen.2024.100610.
9. Takesue, H., Okada, S., Doi, G., Sugiyama, Y., Kusumoto, E., and Ito, T. (2025). Strategic targeting of Cas9 nickase expands tandem gene arrays. Cell Genom. *5*, 100811. https://doi.org/10.1016/j.xgen.2025.100811.
10. Jiao, Y., Li, M., He, X., Wang, Y., Song, J., Hu, Y., Li, L., Zhou, L., Jiang, L., Qu, J., et al. (2023). Targeted, programmable, and precise tandem duplication in the mammalian genome. Genome Res. *33*, 779–786. https://doi.org/10.1101/gr.277261.122.
11. Zhang, R., He, Z., Shi, Y., Sun, X., Chen, X., Wang, G., Zhang, Y., Gao, P., Wu, Y., Lu, S., et al. (2024). Amplification editing enables efficient and precise duplication of DNA from short sequence to megabase and chromosomal scale. Cell *187*, 3936–3952.e19. https://doi.org/10.1016/j.cell.2024.05.056.
12. Ronda, C., Maury, J., Jakočiunas, T., Jacobsen, S.A.B., Germann, S.M., Harrison, S.J., Borodina, I., Keasling, J.D., Jensen, M.K., and Nielsen, A. T. (2015). CrEdit: CRISPR mediated multi-loci gene integration in *Saccharomyces cerevisiae*. Microb. Cell Fact. *14*, 97. https://doi.org/10.1186/s12934-015-0288-3.
13. Verwaal, R., Wang, J., Meijnen, J.P., Visser, H., Sandmann, G., van den Berg, J.A., and van Ooyen, A.J.J. (2007). High-level production of beta-carotene in *Saccharomyces cerevisiae* by successive transformation with carotenogenic genes from *Xanthophyllomyces dendrorhous*. Appl. Environ. Microbiol. *73*, 4342–4350. https://doi.org/10.1128/AEM.02759-06.
14. Azizoglu, A., Brent, R., and Rudolf, F. (2021). A precisely adjustable, variation-suppressed eukaryotic transcriptional controller to enable genetic discovery. eLife *10*, e69549. https://doi.org/10.7554/eLife.69549.
15. Mikkelsen, M.D., Buron, L.D., Salomonsen, B., Olsen, C.E., Hansen, B.G., Mortensen, U.H., and Halkier, B.A. (2012). Microbial production of indolylglucosinolate through engineering of a multi-gene pathway in a versatile yeast expression platform. Metab. Eng. *14*, 104–111. https://doi.org/10.1016/j.ymben.2012.01.006.
16. Orr-Weaver, T.L., and Szostak, J.W. (1983). Multiple, tandem plasmid integration in *Saccharomyces cerevisiae*. Mol. Cell Biol. *3*, 747–749. https://doi.org/10.1128/mcb.3.4.747-749.1983.
17. Plessis, A., and Dujon, B. (1993). Multiple tandem integrations of transforming DNA sequences in yeast chromosomes suggest a mechanism for integrative transformation by homologous recombination. Gene *134*, 41–50. https://doi.org/10.1016/0378-1119(93)90172-Y.
18. Towa, S., Okada, S., and Ito, T. (2025). Catalytically inactive Cas9 attenuates DNA end resection: A potential application for region-restricted random mutagenesis. iScience *28*, 112702. https://doi.org/10.1016/j.isci.2025.112702.
19. Doi, G., Okada, S., Yasukawa, T., Sugiyama, Y., Bala, S., Miyazaki, S., Kang, D., and Ito, T. (2021). Catalytically inactive Cas9 impairs DNA replication fork progression to induce focal genomic instability. Nucleic Acids Res. *49*, 954–968. https://doi.org/10.1093/nar/gkaa1241.
20. Elizondo, B.R., and Saa, P.A. (2025). Complex Kinetic Models Predict β-Carotene Production and Reveal Flux Limitations in Recombinant *Saccharomyces cerevisiae* Strains. ACS Synth. Biol. *14*, 3457–3472. https://doi.org/10.1021/acssynbio.5c00256.
21. Kuivanen, J., Holmström, S., Lehtinen, B., Penttilä, M., and Jäntti, J. (2018). A High-Throughput Workflow for CRISPR/Cas9 Mediated Combinatorial Promoter Replacements and Phenotype Characterization in Yeast. Biotechnol. J. *13*, 1700593. https://doi.org/10.1002/biot.201700593.
22. Brachmann, C.B., Davies, A., Cost, G.J., Caputo, E., Li, J., Hieter, P., and Boeke, J.D. (1998). Designer deletion strains derived from *Saccharomyces cerevisiae* S288C: a useful set of strains and plasmids for PCR-mediated gene disruption and other applications. Yeast *14*, 115–132. https://doi.org/10.1002/(SICI)1097-0061(19980130)14:2<115::AID-YEA204>3.0.CO;2-2.
23. De Coster, W., D'Hert, S., Schultz, D.T., Cruts, M., and Van Broeckhoven, C. (2018). NanoPack: visualizing and processing long-read sequencing data. Bioinformatics *34*, 2666–2669. https://doi.org/10.1093/bioinformatics/bty149.
24. Danecek, P., Bonfield, J.K., Liddle, J., Marshall, J., Ohan, V., Pollard, M.O., Whitwham, A., Keane, T., McCarthy, S.A., Davies, R.M., and Li, H. (2021). Twelve years of SAMtools and BCFtools. GigaScience *10*, giab008. https://doi.org/10.1093/gigascience/giab008.
25. Quinlan, A.R., and Hall, I.M. (2010). BEDTools: a flexible suite of utilities for comparing genomic features. Bioinformatics *26*, 841–842. https://doi.org/10.1093/bioinformatics/btq033.
26. Robinson, J.T., Thorvaldsdóttir, H., Winckler, W., Guttman, M., Lander, E. S., Getz, G., and Mesirov, J.P. (2011). Integrative genomics viewer. Nat. Biotechnol. *29*, 24–26. https://doi.org/10.1038/nbt.1754.
27. Altschul, S.F., Gish, W., Miller, W., Myers, E.W., and Lipman, D.J. (1990). Basic local alignment search tool. J. Mol. Biol. *215*, 403–410. https://doi.org/10.1016/s0022-2836(05)80360-2.
28. Noé, L., and Kucherov, G. (2005). YASS: enhancing the sensitivity of DNA similarity search. Nucleic Acids Res. *33*, W540–W543. https://doi.org/10.1093/nar/gki478.
29. Okada, S., Doi, G., Nakagawa, S., Kusumoto, E., and Ito, T. (2021). Simple-to-use CRISPR-SpCas9/SaCas9/AsCas12a vector series for genome editing in *Saccharomyces cerevisiae*. G3 (Bethesda) *11*, jkab304. https://doi.org/10.1093/g3journal/jkab304.
30. Blount, B.A., Driessen, M.R.M., and Ellis, T. (2016). GC Preps: Fast and Easy Extraction of Stable Yeast Genomic DNA. Sci. Rep. *6*, 26863. https://doi.org/10.1038/srep26863.
31. Takesue, H., Okada, S., and Ito, T. (2025). Long-read plasmid sequencing strategy for evaluating intrinsic instability of tandem gene arrays. MicroPubl. Biol. *2025*. https://doi.org/10.17912/micropub.biology.001582.

Cell Reports Methods
Article

STAR★METHODS

KEY RESOURCES TABLE

REAGENT or RESOURCE	SOURCE	IDENTIFIER
Chemicals, peptides, and recombinant proteins		
17β-Estradiol	Nacalai tesque	Cat# 14541-74
Doxycycline hydrochloride	Apollo scientific	Cat# BID0121
n-Dodecane	Nacalai tesque	Cat# 14205-55
Critical commercial assays		
KOD One® PCR Master Mix (Dye-free 2×PCR Master Mix)	TOYOBO	Cat# KMM-101
KOD SYBR® qPCR Mix	TOYOBO	Cat# QKD-201
Chelex 100 Chelating Resin, biotechnology grade, 100–200 mesh, sodium form	Bio-Rad	Cat# 1432832
Quick-DNA Fungal/Bacterial Miniprep Kit	ZYMO RESEARCH	Cat# D6005
Monarch HMW DNA Extraction Kit for Tissue	NEB	Cat# T3060L
NEB Golden Gate Assembly Kit (BsaI-HF v2)	NEB	Cat# E1601L
NEBuilder HiFi DNA Assembly Master Mix	NEB	Cat# E2621L
Ligation Sequencing Kit	Oxford Nanopore Technologies	SQK-LSK114
Native Barcoding Kit 96 V14	Oxford Nanopore Technologies	SQK-NBD114.96
PromethION Flow Cell (R10.4.1)	Oxford Nanopore Technologies	FLO-PRO114M
Deposited data		
S. cerevisiae S288C reference genome: sacCer3	Saccharomyces Genome Database	https://www.ncbi.nlm.nih.gov/datasets/genome/GCF_000146045.2/
Raw sequence data	This paper	DDBJ BioProject database: PRJDB37536, PRJDB40548
Experimental models: organisms/strains		
S. cerevisiae: Strain background: BY4741 and BY4742	Brachmann et al.[22] 1998	
All other synthetic yeast strains used in this paper, listed in Table S1	This paper	N/A
Oligonucleotides		
All oligonucleotides used in this paper, listed in Table S4	This paper	N/A
Recombinant DNA		
All plasmids used in this paper, listed in Table S2	This paper	N/A
Software and algorithms		
MinKNOW	Oxford Nanopore Technologies	https://community.nanoporetech.com/downloads?from=support
Guppy v6.5.7	Oxford Nanopore Technologies	https://community.nanoporetech.com/downloads?from=support
Dorado v0.7.3	Oxford Nanopore Technologies	https://community.nanoporetech.com/downloads?from=support
NanoPlot	De Coster et al.[23] 2018	https://github.com/wdecoster/NanoPlot
samtools v1.10	Danecek et al.[24] 2021	https://github.com/samtools/samtools
bedtools v2.27.1	Quinlan and Hall[25] 2010	https://github.com/arq5x/bedtools2
Bedgraph_norm_ratio.py	This paper	https://doi.org/10.5281/zenodo.11515696
IGVtools v 2.16.2	Robinson et al.[26] 2011	https://igv.org/

(*Continued on next page*)

Continued

REAGENT or RESOURCE	SOURCE	IDENTIFIER
minialign	Hajime Suzuki	https://github.com/ocxtal/minialign
BLAST	Altschul et al.[27] 1990	https://blast.ncbi.nlm.nih.gov/blast/Blast.cgi
YASS	Noé and Kucherov[28] 2005	https://bioinfo.univ-lille.fr/yass/index.php

EXPERIMENTAL MODEL AND STUDY PARTICIPANT DETAILS

The budding yeast *Saccharomyces cerevisiae* was used as the primary experimental model in the study. The haploid yeast strains BY4741 (*MAT***a** *his3Δ1 leu2Δ0 met15Δ0 ura3Δ0*) and BY4742 (*MAT*α *his3Δ1 leu2Δ0 lys2Δ0 ura3Δ0*) were used as parental strains.[22]

METHOD DETAILS

Yeast strain construction

All yeast strains used in this study are listed in Table S1. They were constructed using standard yeast genetic techniques, including transformation with linearized integrative plasmids (Table S2) or PCR products, as well as genome editing with a previously described vector series.[29]

The gene encoding the artificial transcription factor GEV under the control of the *CUP2* promoter (*pCUP2*) was integrated at the *pCUP2* locus on chromosome VII via plasmid integration with *LEU2* marker. The gene encoding nCas9 (*Streptococcus pyogenes* Cas9^{D10A}), under the control of the *GAL1* promoter (*pGAL1*), was integrated either at the *pGAL1* on chromosome II via plasmid integration with *LEU2* marker or at the *HO* locus on chromosome IV using a previously described genome-editing vector.[29]

In contrast, gRNAs (Table S3) were expressed under the control of *pGAL1* on centromeric plasmids carrying the *URA3* marker (Table S2). Each gRNA was preceded and followed by hammerhead and HDV ribozymes, respectively, to enable self-excision from the primary transcript generated by RNA polymerase II. To co-express three gRNAs, a single vector with two *pGAL1* was used, enabling two gRNAs to self-excise from one mRNA and the third from a separate mRNA.

Two-unit arrays of *CUP1RU* and *ymNGRU*, along with their *HIS3*-interrupted variants, were integrated into the *CUP1* locus on chromosome VIII, the *HO* locus on chromosome IV, or the *X-2* locus on chromosome X,[12] using the genome-editing vector described previously.[29] Following mating between the appropriate **a**-type and α-type haploids, diploid cells were selected for methionine and lysine prototrophy.

We also generated two-unit *tLEU2* arrays, each interrupted by the *crtE*, *crtI*, or *crtYB* gene. These arrays were integrated into the *XI-1,*[15] *X-2*,[12] and *CUP1* loci on chromosomes XI, X, and VIII, respectively. Each carotenogenic gene is under the control of *P7tet.1* promoter and TetR repressor as well as its Tup1-fused variant.[14]

Induction of BITREx

Yeast cells were grown at 30°C overnight in 300 μL of SC−Ura or SC−Ura−Leu medium supplemented with 2% glucose and hygromycin B (Nakalai tesque). On the following day, the OD_{620} of each sample was recorded, and 1–5 μL of the culture diluted up to 1×10^6-fold was inoculated into 300 μL of the fresh medium containing 10 nM β-estradiol. The division number per day was calculated from the change of OD_{620}.

Quantitative PCR

Genomic DNA extracted using the GC prep method[30] was diluted ten-fold with distilled water before qPCR. Each qPCR solution (20 μL) contained 2 μL of diluted DNA, 10 μL of KOD SYBR qPCR Mix (TOYOBO), 0.04 μL of 50× ROX Reference Dye (TOYOBO), 2 pmol each of the forward and reverse primers (Table S4). Each qPCR assay was performed in duplicate, using QuantStudio3 (Applied Biosystems) according to the manufacturer's instructions. The amplification condition was initial denaturation at 98°C for 2 min followed by 40 times iteration of a 3-step thermal cycle composed of 98°C for 10 s, 55°C for 10 s, and 68°C for 30 s. All qPCR runs included 10-fold serial dilutions to generate standard curves. The quantity of target genes was normalized to that of *ACT1*. The copy number of target genes in the standard curves was calibrated by nanopore sequencing results in the BY4741 strain. The CNA/G for each gene was calculated with the formula below: CNA/G = (Copy $\text{number}_{\text{Day 3}}$ − Copy $\text{number}_{\text{Day 0}}$)/Division number.

Nanopore sequencing and data analysis

Genomic DNA was extracted using Monarch HMW DNA Extraction Kit for Tissue (NEB). We avoided vortexing to obtain high molecular weight DNA and used mixing by gentle pipetting with a wide-bore tip. DNA libraries for nanopore whole-genome sequencing were prepared using the ligation sequencing kit SQK-LSK114 and the native barcoding kit SQK-NBD114 (Oxford Nanopore Technologies) according to the manufacturer's instructions. We modified the protocol of the ligation sequencing kit as follows: DNA

fragmentation, omitted; duration of the enzymatic repair steps at 20°C and 65°C, both extended from 5 min to 30 min; and the duration of the ligation step, extended from 10 min to 30 min; incubation time for elution with 0.4× AMPure XP, extended from 10 min to 20 min. The library was sequenced with the flowcell FLO-PRO114M R10.4.1 using the PromethION 2 Solo sequencer (Oxford Nanopore Technologies). MinKNOW software was used to control the PromethION device. The run time was set to 72 h. Base calling was performed using Guppy v6.5.7 and Dorado v0.7.3. The assessment of sequencing data was performed using NanoPlot.[21]

We used nanopore sequencing data in FASTQ format and mapped reads to the S288c reference genome (version R64-2-1, http://sgd-archive.yeastgenome.org/sequence/S288C_reference/genome_releases/S288C_reference_genome_R64-2-1_20150113.tgz) using SAMtools[24] and BEDtools,[25] and then normalized read count of each nucleotide was calculated using Bedgraph_norm_ratio.py (https://doi.org/10.5281/zenodo.11515696). Data were visualized with the IGV.[26] To eliminate the effect of read clipping and achieve a more accurate estimation of repeat unit number, we collected all reads containing the repeat unit using minialign (https://github.com/ocxtal/minialign). We then used the reference sequence of a target gene as a query in a BLAST[26] search against the collected reads and estimated the copy number based on the number of BLAST hits, as described previously.[9,31]

We used nanopore sequencing data in FASTA format to draw dot plots using YASS.[28] We first selected reads spanning the entire array using 1-kb upstream and downstream sequences of the target array as queries of minialign (https://github.com/ocxtal/minialign) and then used these reads as the first input sequence for YASS. As the second input, we used the reference sequence of the repeat unit. By manually counting the diagonal lines in each dot plot, we determined the copy number of the repeat unit.

Extraction of β-carotene

Yeast cells were grown at 30°C overnight in 2 mL of YPA medium supplemented with 2% glucose and 50 μg/mL doxycycline (Apollo scientific). On the following day, cells were harvested by transferring the culture medium into 2-mL microcentrifuge tubes followed by centrifugation at 15,000 rpm for 1 min. The supernatant was removed, and the pellet was resuspended in 100 μL of 5% Chelex solution (Bio-Rad) supplemented with glass beads. Cells were disrupted using a Disruptor Genie (Scientific Industries) for 5 min. Subsequently, 300 μL of *n*-dodecane (Nacalai tesque) was added, and the mixture was vortexed with the Disruptor Genie for 15 min to extract β-carotene into the organic phase. Samples were centrifuged at 15,000 rpm for 1 min, and the upper phase was transferred to a 96-well plate. Absorbance was measured at 405, 450, 492, and 620 nm using a Byonoy Absorbance 96 Plate Reader (Byonoy).

QUANTIFICATION AND STATISTICAL ANALYSIS

All experiments were conducted with three independent biological replicates ($n = 3$) per strain, as detailed in the legends for Figures 2 and 3. Gene copy numbers were quantified by qPCR and normalized to those of *ACT1*, and cell densities were measured by spectrophotometry (OD_{620}); both measurements were performed at day 0 and day 3.

Statistical analysis was performed using SciPy (1.16.3) in Python (3.13.9). For comparisons between two groups, unpaired two-tailed Student's t-tests were used to determine statistical significance, assuming normal distribution, with $p < 0.05$ considered statistically significant.

Data visualization was conducted using the matplotlib (3.10.6) and seaborn (0.13.2) libraries in Python (3.13.9). Line graphs illustrating the copy number alterations from day 0 to day 3 represent the mean values, with the SD shown as shaded areas. Bar graphs for copy numbers at day 3 and the mean CNA/G values display the mean ± SD, with individual data points overlaid to ensure data transparency.

Cell Reports Methods, Volume *6*

Supplemental information

Parallel gene amplification by Cas9 nickase for generating functionally heterogeneous cell populations

Hiroaki Takesue, Satoshi Okada, and Takashi Ito

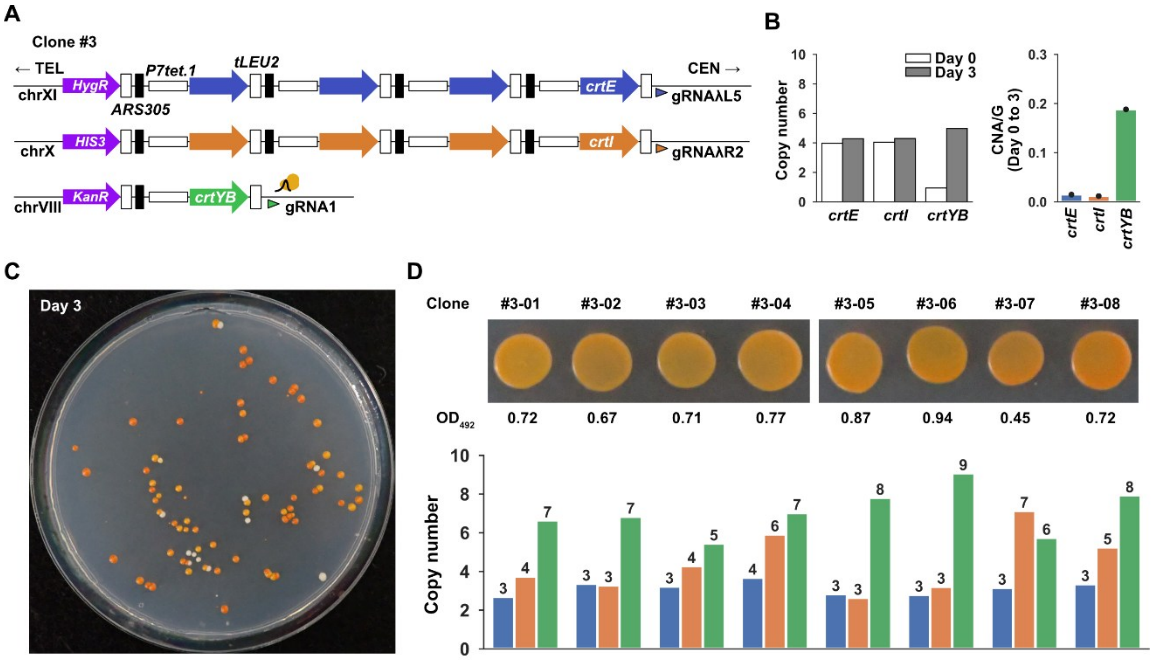

Figure S1. Independent generation of elite clones by simplex BITREx, related to Figure 4

(A) Genetic architecture of the parental strain clone #3. This parental strain had already acquired four copies each of *crtE* and *crtI* at the initial strain construction phase using the standard genome editing. Accordingly, only gRNA1 was expressed in clone #3 to induce the selective amplification of *crtYB*.

(B) Copy number alterations of carotenogenic genes. Clone #3 was subjected to simplex BITREx for *crtYB* in the presence of β-estradiol. Copy numbers of each target gene at day 0 and day 3 were quantified by qPCR (left and middle panels), and the corresponding CNA/G values were calculated (right panel).

(C) Colonies on Dox-containing agar plates. To cease BITREx and induce β-carotene synthesis, the 3-day culture of clone #3 was spread on agar plates containing Dox but lacking β-estradiol.

(D) Analysis of representative elite clones. The 3-day culture of clone #3 was spread on agar plates containing Dox but lacking β-estradiol to cease BITREx and induce β-carotene synthesis. Top panel: Representative eight clones are shown as patches alongside their corresponding OD_{492} values of the cell extract, which serve as a proxy for β-carotene yield. Bottom panel: Bar graphs indicate the copy numbers of the three carotenogenic genes in these eight clones, as estimated from nanopore sequencing data.

A

Clone	Day 0			Day 4			
	Copy number value			Generation	Copy number value		
	crtE	crtI	crtYB		crtE	crtI	crtYB
#1-01	10 (1.00)	1 (1.00)	BLQ (N.C.)	30.5	15 (1.41)	1 (1.31)	BLQ (N.C.)
#1-02	15 (1.00)	BLQ (N.C.)	5 (1.00)	30.4	15 (1.00)	BLQ (N.C.)	5 (1.06)
#1-03	3 (1.00)	BLQ (N.C.)	18 (1.00)	30.8	3 (0.92)	BLQ (N.C.)	13 (0.75)
#1-04	14 (1.00)	BLQ (N.C.)	3 (1.00)	28.6	16 (1.15)	BLQ (N.C.)	5 (1.67)
#1-05	32 (1.00)	18 (1.00)	1 (1.00)	30.8	20 (0.63)	18 (1.04)	1 (1.27)
#1-06	45 (1.00)	7 (1.00)	1 (1.00)	29.9	45 (1.01)	8 (1.09)	1 (1.62)
#1-07	25 (1.00)	1 (1.00)	2 (1.00)	28.6	27 (1.10)	1 (1.16)	3 (1.49)
#1-08	19 (1.00)	1 (1.00)	1 (1.00)	28.4	23 (1.21)	2 (2.73)	2 (2.39)
#1-09	12 (1.00)	15 (1.00)	6 (1.00)	28.1	12 (1.04)	17 (1.15)	9 (1.58)
#1-10	20 (1.00)	1 (1.00)	5 (1.00)	28.5	21 (1.05)	1 (1.51)	7 (1.44)
#1-11	9 (1.00)	1 (1.00)	10 (1.00)	28.3	16 (1.84)	2 (2.73)	19 (1.87)
#1-12	18 (1.00)	1 (1.00)	5 (1.00)	28.6	22 (1.18)	3 (3.65)	8 (1.76)
#1-13	16 (1.00)	4 (1.00)	10 (1.00)	30.5	15 (0.95)	8 (1.76)	12 (1.24)
#1-14	7 (1.00)	17 (1.00)	3 (1.00)	31.4	8 (1.12)	21 (1.21)	4 (1.33)
#1-15	13 (1.00)	37 (1.00)	9 (1.00)	31.2	15 (1.76)	29 (0.78)	11 (1.27)
#1-16	4 (1.00)	4 (1.00)	7 (1.00)	29.2	5 (1.21)	7 (1.70)	11 (1.50)

B

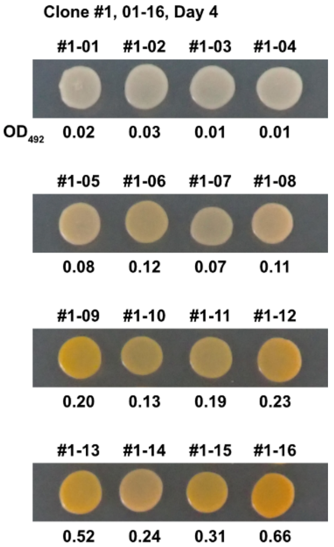

Figure S2. Stability of expanded arrays of carotenogenic genes, related to Figure 4

(A) Stability of copy numbers for the three carotenogenic genes during 4-day cultivation. Copy-number retention in the 16 clones (originally shown in Figure 4E) was monitored via qPCR during passaging without β-estradiol. The estimated generation numbers, derived from OD_{620} values, are provided for each time point. Values in parentheses represent relative copy numbers normalized to those at day 0. BLQ, below the lower limit of quantification; N.C., not calculated.

(B) Maintenance of production phenotypes. Cell spots of the 16 clones after 4-day cultivation on Dox-containing agar plates are shown alongside the corresponding OD_{492} values of the cell extracts.
